# *Paussus (Scaphipaussus) zhouchaoi* sp. n., a new myrmecophilous species from China (Coleoptera, Carabidae, Paussinae, Paussini)

**DOI:** 10.3897/zookeys.663.11635

**Published:** 2017-03-28

**Authors:** Cheng-Bin Wang

**Affiliations:** 1 Bin Insect Taxonomy Studio, No.16, Xizhaosi Street, Dongcheng District, Beijing 100061, P. R. China; 2 Department of Ecology, Faculty of Environmental Sciences, Czech University of Life Sciences Prague, Kamýcká 129, CZ-165 21 Praha 6, Czech Republic

**Keywords:** Carabidae, China, new species, Paussinae, Paussini, *Paussus*, *Scaphipaussus*, taxonomy

## Abstract

A new species of flanged bombardier beetles is described from Jiangxi and Sichuan, China, Paussus (Scaphipaussus) zhouchaoi **sp. n.** (Coleoptera, Carabidae, Paussinae, Paussini). All the type specimens were collected from colonies of the host ant *Pheidole* sp. (Hymenoptera, Formicidae, Myrmicinae). Important morphological characters of the new species are illustrated by color plates.

## Introduction

The obligate myrmecophilous genus *Paussus* Linné, 1775 is the most speciose genus of Paussinae (Coleoptera, Carabidae) with members distributed in Afrotropical, Oriental, southwest and southeast Palearctic and Madagascan Regions. Recently, Robertson and Moore (2016) excellently revised *Paussus* Linné, subdividing and delineating this genus into three series and 10 subgenera:

*Paussus* I series (*Bohemanipaussus* Luna de Carvalho, 1982; *Bathypaussus* Wasmann, 1929; *Edaphopaussus* Kolbe, 1920);

*Paussus* II series (*Paussus* Linné, 1775; *Klugipaussus* Kolbe, 1927; *Scaphipaussus* Fowler, 1912; *Hylotorus* Dalman, 1823; *Anapaussus* Wasmann, 1929);

*Paussus* III series (*Lineatopaussus* Kolbe, 1928; *Shuckardipaussus* Kolbe, 1938).

However, for the fauna of China, only eleven species were recorded (Luna de Carvalho 1989, Maruyama 2016, Nagel 2003, Robertson and Moore 2016):

*Paussus* series II: subgenus Paussus Linné, 1775:

*P. brancuccii* Nagel, 2016 (Guangxi);

*P. kjellanderi* Luna de Carvalho, 1965 (Jiangsu, Taiwan).

*Paussus* series II: subgenus Scaphipaussus Fowler 1912:

*P. bowringii* Westwood, 1850 (Hong Kong);

*P. formosus* Wasmann, 1912 (Taiwan);

*P. hystrix* Westwood, 1850 (Hong Kong, Jiangsu, Sichuan);

*P. jengi* Maruyama, 2016 (Taiwan).

*Incertae sedis*:

*P. elongatus* Kanô, 1930 (Taiwan);

*P. horikawae* Kanô, 1930 (Taiwan);

*P. minor* Shiraki, 1907 (Taiwan);

*P. sauteri* Wasmann, 1912 (Taiwan);

*P. jousselini* Guérin-Méneville, 1836 (Hong Kong, Hunan).

In this paper, a new species from Jiangxi Province and Sichuan Province of China is described and illustrated: Paussus (Scaphipaussus) zhouchaoi sp. n. All the type specimens were collected from colonies of the host ant *Pheidole* sp. (Hymenoptera, Formicidae, Myrmicinae).

## Material and methods

**Figure 1.**
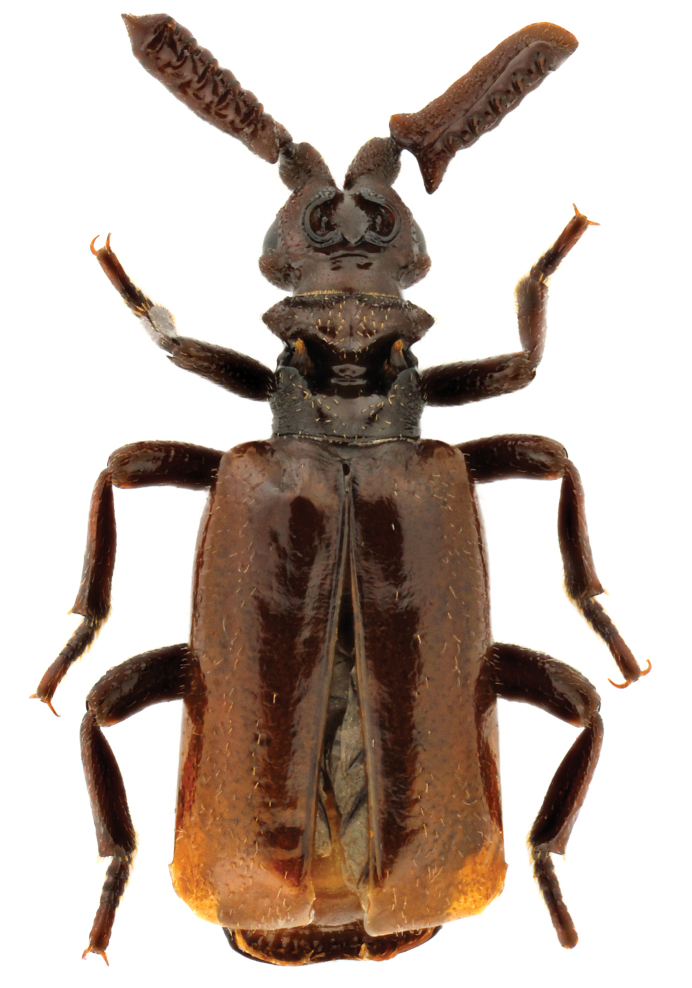
Habitus of Paussus (Scaphipaussus) zhouchaoi sp. n. (holotype, ♂; dorsal view).

Specimens were relaxed and softened in a hot saturated solution of potassium hydroxide for three minutes, and then transferred to distilled water to rinse the residual potassium hydroxide off and stop any further bleaching. The softened specimens were moved into glycerin and dissected there to observe morphological details. After examination, the body parts were mounted on a glass slip with Euparal Mounting Medium for future studies. Habitus photograph of Figure 1 was taken using a Canon macro photo lens MP-E 65mm on a Canon 550D. Observations and measurements were performed using a Zeiss Axio Zoom.V16 motorized stereo zoom microscope (magnification up to ×270). Color photographs were taken with a Zeiss AxioCam MRc 5 and the final deep focus images were created with the stacking software Helicon Focus 5.3. Adobe Photoshop CS6 was used for image post-processing. The morphological terminology follows Nagel (1987) and Robertson and Moore (2016).

The material examined for this study is deposited in the following collections and museums:

**BITS** Bin Insect Taxonomy Studio, Beijing, China

**SNUC** Insect Collection of Shanghai Normal University, Shanghai, China

Measurement criteria in millimeters (mm) are used as follows:

**Body length**: axial length from the anterior apex of clypeus to the elytral apex.

**Head length**: axial length from the anterior apex of clypeus until the constriction of neck.

**Head width**: maximum width of head (including eyes or temples).

**Eye length**: shortest diameter of eye between anterior and posterior margins.

**Gena length**: shortest distance between antennal insertion and anterior margin of eye.

**Pronotal length**: axial length of pronotum.

**Pronotal width**: maximum width of pronotum.

**Pronotal anterior part length**: axial length from the anterior margin of pronotum to the anterior edge of transverse pronotol cleft.

**Pronotal anterior part width**: maximum width of pronotal anterior part.

**Pronotal posterior part length**: length from the level of forefront after transverse pronotol cleft to the posterior margin of pronotum.

**Pronotal posterior part width**: maximum width of pronotal posterior part.

**Elytral length**: length from the basal border of elytra to its apex along suture.

**Elytral width**: width across the middle of two elytra combined together.

## Results

### Genus *Paussus* Linné, 1775

Vernacular name: 棒角甲属

### Subgenus Scaphipaussus Fowler 1912

Vernacular name: 舟棒角甲亚属

#### Paussus (Scaphipaussus) zhouchaoi sp. n.

Taxon classificationAnimaliaORDOFAMILIA

http://zoobank.org/B05E8634-5A8D-407F-97FD-FF6BAB547BA5

**Figure 2.**
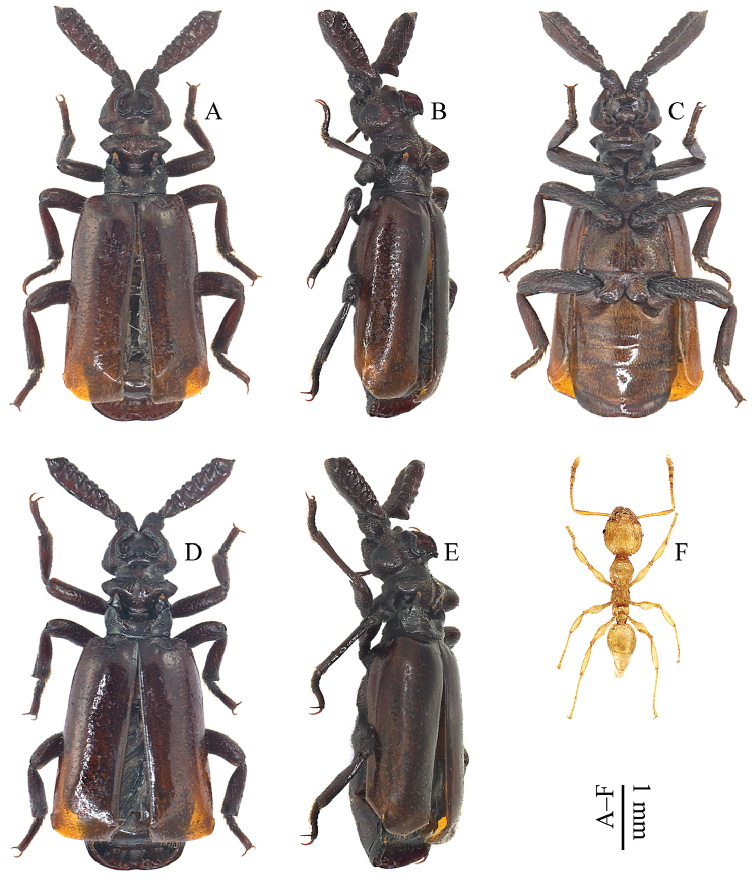
Habitus of Paussus (Scaphipaussus) zhouchaoi sp. n. (**A–C** holotype, ♂ **D–E** paratype, ♀) and its host ant (**F** *Pheidole* sp., minor worker) **A, D, F** dorsal view **B, E** dorsolateral view **C** ventral view.

**Figure 3.**
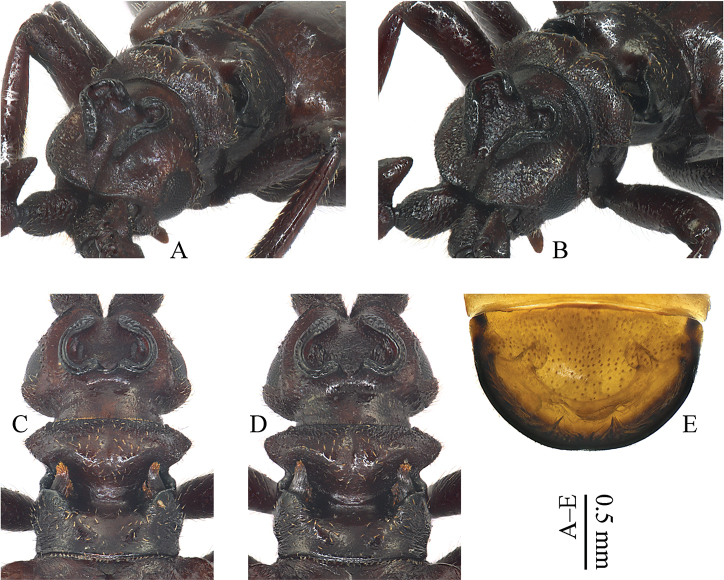
Paussus (Scaphipaussus) zhouchaoi sp. n. (**A, C, E** holotype, ♂ **B, D** paratype, ♀). **A–D** heads and pronota **E** pygidium **A–B** oblique anterodorsal view **C–E** dorsal view.

**Figure 4.**
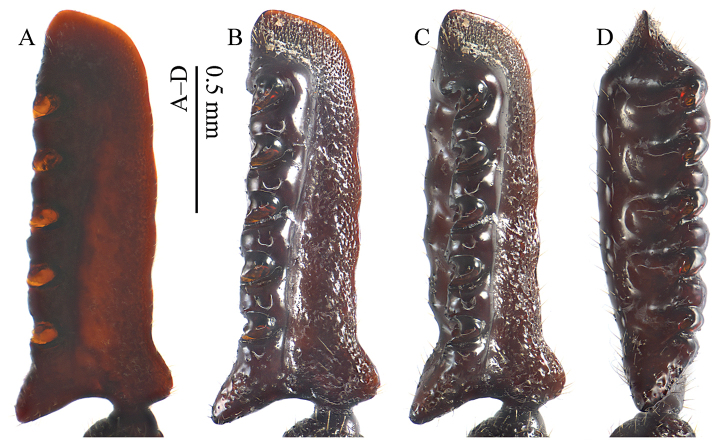
Antenna of Paussus (Scaphipaussus) zhouchaoi sp. n. (holotype, ♂). **A** dorsal view, in ethanol solution **B** dorsal view **C** dorsoposterior view **D** posterior view.

**Figure 5.**
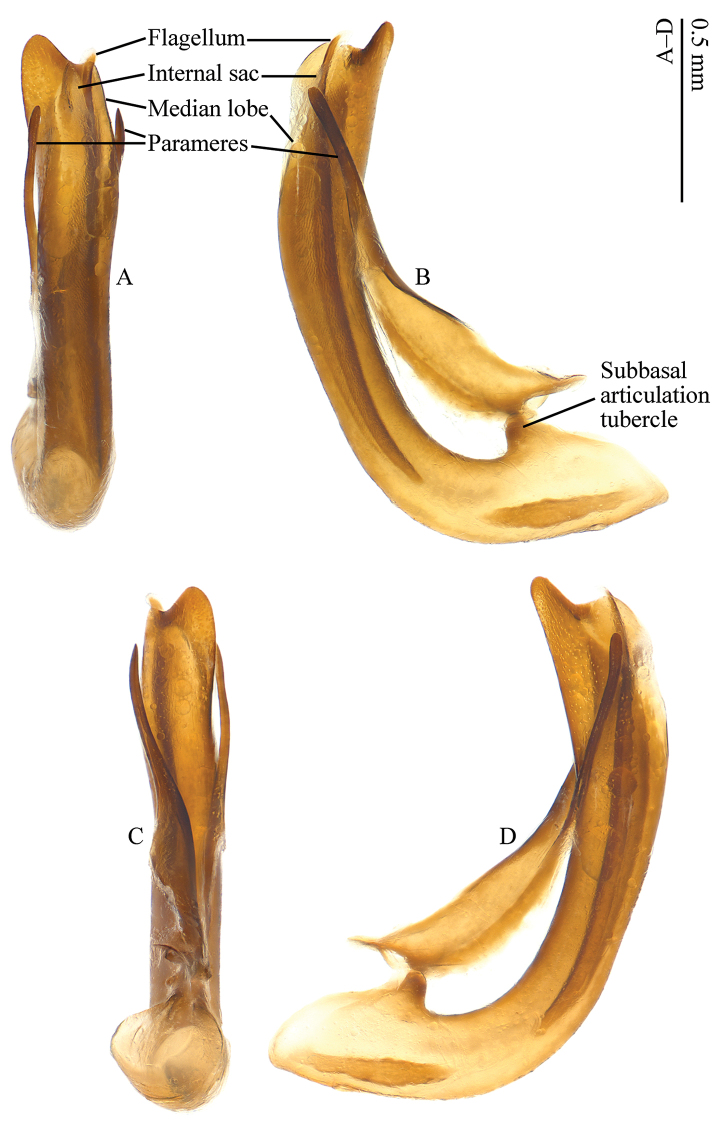
Aedeagus of Paussus (Scaphipaussus) zhouchaoi sp. n. (holotype, ♂). **A** dorsal view **B** left lateral view **C** ventral view **D** right lateral view.

**Figure 6.**
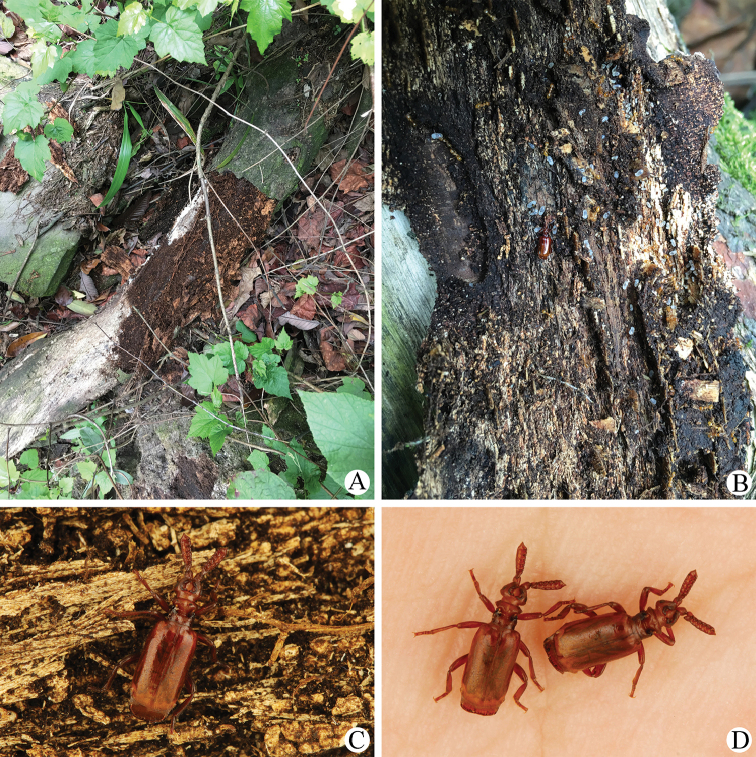
Field observations (Sichuan: Lingyanguanyinshan scenic area) of Paussus (Scaphipaussus) zhouchaoi sp. n. (Photoed by Li He) **A** biotope **B** the rotten deadwood inhabited by P. (S.) zhouchaoi sp. n. and its host ants (*Pheidole* sp.) **C** P. (S.) zhouchaoi sp. n. on the rotten deadwood **D** P. (S.) zhouchaoi sp. n. on the palm of the collector Li He.

Vernacular name: 周超棒角甲 Figs 1 ; 2A–E ; 3 ; 4 ; 5 ; 6B–D

##### Material examined.

**Holotype**: ♂, CHINA, Sichuan: Chengdu City, Dujiangyan, Zipingpu Town, Lingyanguanyinshan scenic area (灵岩观音山风景区), 29.IV.2016, 31.02956N, 103.61651E, alt. 1210 m, ant colony under deadwood bark, leg. Chao Zhou & Li He (BITS). **Paratypes**: 1♀, same data as holotype (BITS); 1♂, CHINA, Jiangxi: Yichun City, Mingyueshan (明月山), 27°35'25"N, 114°17'02"E, alt. 1600 m, 22.X.2013, Zhong Peng leg. [from a colony of *Pheidole* sp.] (SNUC).

##### Diagnosis.

This new species is allocated to the subgenus Scaphipaussus Fowler 1912 according to the groups key of Robertson & Moore (2016). Within this subgenus the new species is unique with regard to the combination of the following characters: body lustrous, scatteredly and shortly pubescent, seeming hairless to the naked eyes; head vertex distinctly excavated, laterally bordered by auriculate costae; scape longer than wide, cylindrical; fused flagellum elongated subtriangular, with dorsal margin of outer side with five robust teeth and five deep incisions, while ventral margin of that with five weak protrusions and five weak emarginations; pronotum wider than long, width/length = 1.22, as wide as head, (anterior part width)/(posterior part width) = 1.10, anterior part laterally angulate, posterior part with lateral margins roundly protruded in the apical 3/4 and obliquely substraight in the basal 1/4; elytra not bearing lateral trichome fringes; legs robust; pygidium with posterior dorsal margin distinctly upturned and explanate, marginal trichome fringe dense.

##### Description.

***Male holotype***. Medium size, body 4.83 mm long. Length (mm) of different body parts: head (0.68) : pronotum (0.91) : pronotal anterior part (0.40) : pronotal posterior part (0.43) : elytra (3.21) : pronotum-elytra (4.12); width (mm): head (1.13) : pronotum (1.11) : pronotal anterior part (1.11) : pronotal posterior part (1.01) : elytra (1.88). (Pronotum-elytra length)/(elytral width) = 2.20.

*Body* (Figs 1; 2A–C) oblong and overall appearance lustrous due to lack of punctures or microsculptures on the majority of body parts; unicolor, mostly brown, with elytra lighter and pronotal posterior part darker (the body color is much lighter when the species is alive (Fig. 6C–D) or the specimens are newly collected); integument scatteredly pubescent with short, thin, yellowish setae except pygidium, and the setae on pronotum are distinctly thicker and shorter.

*Head* (Fig. 3A, C) subglobular, width/length = 1.67, vertex high and dorsal parts strongly inclined; clypeus indented, depressed at centre and with frontal ridges well demarked; median frontal suture short and dark, not extending to anterior margin of eye; vertex distinctly and broadly excavated, bordered by a raised, auriculate, double-walled costa at each side; the two costae are separated at their tops by a distinct groove; in addition, one straight and short costa sits in the basal corner of each auriculate costa; eyes reniform, small and less prominent; temples narrow but distinct, slightly projecting laterally beyond eyes in dorsal view; (eye length)/(gena length) = 1.23; head surface rugosely and contiguously punctate, especially along median frontal suture, while central excavation glabrous and smooth; neck narrow, strongly constricted.

*Mouthparts* of “closed” type, adjacent to the underside of head. Labrum transverse and rectangular. Palpifer exposed. Maxillary palpomere II large, broad and compressed, almost as wide as long, wider than 2× width of palpomere III and longer than palpomeres III and IV combined, with mesal margin more or less rounded; palpomere III only slightly wider than palpomere IV; palpomere IV tapering apically and slightly longer than palpomere III. Palpiger exposed. Labial palpomere II with socket for palpomere III positioned along midline; palpomere III narrow, slender, fusiform and slightly compressed, length/width = 3.43, longer than 2× length of palpomeres I and II combined. Ligula large and broad, with apical margin broadly rounded at middle. Gula with width/length (at narrowest point) = 0.42.

*Antenna* (Fig. 4A–D): scape longer than wide, cylindrical, surface rugose, without trichome brush on inner anterior margin. Pedicel vestigial ring-shaped. Fused flagellum elongated subtriangular, 2.59 times as long as wide; dorsal surface with a shallow longitudinal furrow, ending at the level of the most apical incision; ventral surface slightly convex; inner side broadly and weakly undulate; apical side obliquely truncate and rounded at tip; both apical side and inner side (especially along apical half) marginate and the marginal band rugosely punctate; outer side longitudinally excavated from outer basal angle to short distance before apex; dorsal margin of outer side with five robust teeth and 5 deep incisions; ventral margin of outer side with five weak protrusions and five weak emarginations; inner basal corner roundly protruded; outer basal angle large, prominent and extended, without trichome; basal side between insertion and outer basal angle simply emarginate without traces of indentations.

*Pronotum* (Fig. 3A, C) wider than long, width/length = 1.22, as wide as head, widest at lateral angulations of anterior part; transversally cleft, anterior part almost as long as posterior part, (anterior part width)/(posterior part width) = 1.10; anterior part moderately raised, strongly divided by a longitudinal groove, weakly edged behind and laterally angulate; posterior part with lateral margins roundly protruded in the apical 3/4 and obliquely substraight in the basal 1/4, medially with a deep, wide and longitudinal furrow towards scutellum; transverse furrow with trichome-bearing glandular openings at the furthest lateral ends; trichomes dark yellow, not much contrasting with the brown pronotum, (distance between trichomes)/(trichome width) = 3.38; median excavation glabrous and smooth, both sides of excavation equally rugose.

*Scutellum* ligulate, wider than long, surface densely punctate.

*Elytra* oblong, length/width = 1.71; humeri hardly demarcated; surface smooth and lustrous, without punctures, merely sculptured with micropores; areas along suture devoid of pubescence. Metathoracic wings fully developed.

*Legs* robust, with smooth surfaces. Tibiae compressed; protibiae straight, mesotibiae slightly sinuate, metatibiae almost straight; metatibiae broader than meso- and protibiae; tibial spurs absent; pubescence denser on apical part of all tibiae. Tarsi with tarsomeres I–IV subequal in length, with apical margins entire, dorsally straight or inconspicuously emarginate; all tarsomeres without adhesive pads but loosely setose on lateral parts of ventral surfaces.

*Stridulatory organ* present: scraper as a curved row of transverse spines on abdominal ventrite I, and file present at inner base of metafemur.

*Pygidium* (Fig. 3E) with disc shining, only sparsely covered with microtrichiae; posterior dorsal margin distinctly upturned and explanate; marginal trichome fringe dense.

*Aedeagus* as shown in Figs 5A–D: median lobe elongate, slender and arcuate, apex distinctly emarginate; subbasal articulation tubercle well developed; parameres slender, apical parts narrow, apex rounded and devoid of setae; strut present, embedded in membraneous tissue in the basal part of median lobe.

***Female paratype*** (Figs 2D–E; 3B, D). Sexual dimorphism weak, no distinct differences between shape of eyes, shape of costae at head vertex, shape and microstructure of fused flagellum, curvature of metatibiae. The abdomen of the female paratype, including female genitalia, was poured into wash basin when the present author working in a drunken state.

##### Host ant.

All the type specimens were collected from colonies of the host ant *Pheidole* sp. (Hymenoptera, Formicidae, Myrmicinae). The male holotype and the female paratype from Sichuan Province were collected from a *Pheidole* colony under deadwood bark. Two *Pheidole* sp. minor workers (Fig. 2F) glued to a card and pinned together with the holotype.

##### Field observations.

Biotope in Lingyanguanyinshan scenic area (Sichuan) as shown in Fig. 6A–B.

##### Etymology.

The specific epithet is dedicated to Mr. Chao Zhou (Chengdu, Sichuan, China), one of the collectors of this new species and a good amateur obsessing with beetles.

##### Distribution.

China (Jiangxi, Sichuan).

##### Remarks.

Maruyama (2016) partially revised the *Paussus hystrix* group (to which unequivocally the new species belongs) and described 19 species from East Asia and Southeast Asia. Comparing with these species, Paussus (Scaphipaussus) zhouchaoi sp. n. seem to be hairless to the naked eye and it also can be distinguished by the combination of the characters in the above paragraph of Diagnosis.

## Supplementary Material

XML Treatment for Paussus (Scaphipaussus) zhouchaoi
